# Conservative and Surgical Treatment of Talar Fractures: A Systematic Review and Meta-Analysis on Clinical Outcomes and Complications

**DOI:** 10.3390/ijerph18168274

**Published:** 2021-08-04

**Authors:** Babak Saravi, Gernot Lang, Robert Ruff, Hagen Schmal, Norbert Südkamp, Sara Ülkümen, Jörn Zwingmann

**Affiliations:** 1 Department of Orthopedics and Trauma Surgery, Medical Center, Faculty of Medicine, Albert-Ludwigs-University of Freiburg, 79106 Freiburg, Germany; gernot.lang@rkk-klinikum.de (G.L.); robert.ruff@diak-fr.de (R.R.); hagen.schmal@uniklinik-freiburg.de (H.S.); norbert.suedkamp@uniklinik-freiburg.de (N.S.); sara.uelkuemen@jupiter.uni-freiburg.de (S.Ü.); Joern.Zwingmann@oberschwabenklinik.de (J.Z.); 2 Department of Spine Surgery, Loretto-Krankenhaus Freiburg, 79100 Freiburg, Germany; 3 Department of Orthopedics and Trauma Surgery, St. Elisabeth Hospital Ravensburg, 88212 Ravensburg, Germany

**Keywords:** talus, ankle fractures, ankle injuries, osteoarthritis, osteonecrosis, systematic review

## Abstract

The integrity of the talus is crucial for the physiologic function of the feet. The present study sought to summarize the available evidence on clinical outcomes and complications following conservative and surgical treatment of talar fractures. We systematically searched Medline via OVID to find relevant studies with a follow-up of at least six months. Hereafter, the success and complication rates were extracted and analyzed in a random effects proportion meta-analysis. Complications were defined as avascular bone necrosis (AVN) and posttraumatic osteoarthritis (OA). Additionally, a subgroup analysis was performed for fracture localization (talar neck fractures (TN) and combined talar body/neck fractures (TN/TB)) and severity of the fracture. The quality of the included studies was assessed utilizing the Coleman Methodology Score (CMS). A total of 29 retrospective studies, including 987 fractures with a mean follow-up of 49.9 months, were examined. Success rates were 62%, 60%, and 50% for pooled fractures, TN, and TN/TB, respectively. The overall complication rate for AVN was 25%. The rate was higher for TN (43%) than TN/TB (25%). Talar fractures revealed a 43% posttraumatic osteoarthritis (OA) rate in our meta-analysis. Success rates showed an association with fracture severity, and were generally low in complex multi-fragmentary fractures. The mean CMS was 34.3 (range: 19–47), indicating a moderate methodological quality of the studies. The present systematic review on clinical outcomes of patients undergoing conservative or surgical treatment for talar fractures reveals a lack of reliable prospective evidence. Talar fractures are associated with relatively poor postoperative outcomes, high rates of AVN, and posttraumatic osteoarthritis. Poor outcomes revealed a positive association with fracture severity. Prospective studies investigating predictors for treatment success and/or failure are urgently needed to improve the overall quality of life and function of patients undergoing surgical treatment due to talar fractures.

## 1. Introduction

Talar fractures account for 0.3% of all fractures and 3.4% of foot fractures [1,2]. The integrity of the talus is crucial to the normal function of the ankle, subtalar, and transverse tarsal joints. Injuries to the head, neck, or body of the talus can disrupt the physiologic movement of these joints, potentially leading to chronic pain, loss of motion, and deformity. Typical findings after incorrectly healed talus fractures are axial deviations, in particular varus malalignment, posttraumatic osteoarthritis of the upper and lower ankle joints, symptomatic pseudarthrosis, impingement of the posterior tibial tendon or tarsal tunnel syndrome due to bony prominences, and, finally, the collapse of the talar body [3]. One of the most common causes of central talar fractures is a fall from a great height [1]. The typical trauma mechanism of talar neck fractures (contributing to more than 50% of all talar fractures) involves forced dorsiflexion of the foot combined with extensive axial forces. Consequently, the neck of the talus is sheared between the anterior edge of the tibia and the sustentaculum tali. In contrast, forced plantar flexion combined with a rotational component at the time of violence is held responsible for talar body fractures (around 20% of all talar injuries) [4]. The rare talar head fractures (less than 10% of all talar fractures) usually occur when the midtarsal (Chopart) joint is involved, and are caused by forced abduction or adduction of the forefoot with simultaneous rotation of the rear foot [5]. Notably, 13–15% of all talar fractures are open fractures [6]. Concomitant injuries are found in nearly half of all talar fractures [4,7]. Severe soft tissue damage occurs in approximately 15% of cases [6]. Additional regional injuries include malleolar fractures (in up to 44% of fracture cases), injuries to the calcaneus (in 11–18% of fracture cases), and concomitant metatarsal fractures (in up to 18% of fracture cases) [8,9].

The talar body is mainly supplied by an anastomosis between the canalis tarsi artery and the sinus tarsi artery. The vessels enter the talar body at the level of the talar neck and run from distal to proximal. If this anastomosis ruptures due to dislocated fractures of the talar neck, the risk of bone necrosis is significantly increased [10]. Additionally, the deltoid branch is discussed as an important vessel that revascularizes the talus from the medial side after fractures and should, therefore, be spared in medial surgical access [10,11,12]. The extensive cartilaginous surface of the talus affects blood supply and leads to a high risk of posttraumatic damage, such as avascular bone necrosis (AVN) and posttraumatic osteoarthritis [11]. AVN is reported to occur in up to 50% of central fractures and dislocations [13]. Although the necrosis rate is reported to vary considerably, there seems to be a correlation with the initial degree of dislocation. AVN is found in 0–24% of Hawkins type I fractures, whereas 33–100% occur in Hawkins type III and IV fractures [11,14]. Non-displaced fractures of the talar body (Marti type II) are associated with AVN in 5–44% of cases, and displaced talar body fractures (Marti types III and IV) can result in AVN in up to 50% of patients [15]. With open fractures, the risk of AVN appears to be increased [16,17].

The rates for posttraumatic osteoarthritis after talar neck and body fractures vary between 16% and 100% [16,18]. One possible explanation could be the lack of standardized diagnostic criteria and different follow-up periods in the literature. The rate of posttraumatic osteoarthritis seems to increase over time. However, not all cases become symptomatic. A clear link between the severity of the fracture and AVN could not be established to date [17,19]. A malalignment of the talar neck, which leads to significant load-bearing properties in the ankle and subtalar joints, or remaining unevenness in the joint surface, may cause poorer clinical results. Therefore, these conditions should be considered as pre-arthritic triggers [16,20,21,22]. Other reported postoperative complications are dermal necrosis (11%) and infections (3–8%) [6,16,19,23]. Septic necrosis of the talar body is a severe complication, not infrequently requiring partial or total removal of the talus [24,25].

The general goal of therapy is early restoration of the anatomical situation. The congruence of the ankle and subtalar joints should be restored promptly by optimizing the remaining blood supply to reduce the risk of avascular necrosis [2]. Non-displaced fractures of the talar body can be treated conservatively with a lower leg orthosis up to 16 weeks [6,14,26,27,28,29]. However, osteoarthritis of the ankle joint and/or lower ankle joint can also occur in such low-grade fractures [1]. Surgical repositions and fixations are required for high-graded fractures and dislocations. Nevertheless, early mobilization and physiotherapy are recommended for functional rehabilitation. Fractures of the talar head have a more favorable prognosis than fractures of the body or neck due to the limited blood supply [1,30]. Moreover, therapeutical outcomes vary widely, and are frequently related to the degree of initial fracture displacement. Minimally displaced fractures can be hard to diagnose and might miss early treatments, followed by poor outcomes [31]. Peripheral and non-dislocated central talar fractures are often misdiagnosed as distortions in the upper or lower ankle [32].

There is controversy regarding the optimal therapeutic approaches for talus fractures. Specifically, the rate of posttraumatic osteoarthritis, secondary interventions, avascular necrosis, and the effect of the interval until surgical treatment on the factors mentioned need to be further explored [33,34]. Clinical outcomes depend on the initial degree of dislocation, joint involvement, and soft tissue damage. However, clinical reports vary considerably. Results with good and excellent clinical outcomes for talar neck fractures range from 40–100%, 32–80%, and 15–55% for Hawkins I fractures, Hawkins II fractures, and Hawkins III fractures, respectively. These high variation ranges may be caused by different distributions of concomitant injuries [32]. In the most extensive study to-date on talar body fractures, 39 of 66 patients (59%) complained of temporary or persistent symptoms [35]. Furthermore, the authors reported posttraumatic osteoarthritis in eight out of nine (88%) conservatively treated fractures.

Available reviews on this topic focused solely on talar neck fractures [33,36,37] or talar head fractures [38]. Considering the variability in the literature and the development of new therapeutic options, an updated comprehensive review focusing on both talar neck and body fractures that summarizes the current status of clinical outcomes of talar fractures is warranted. The inclusion of both conservative and surgical treatments, such as multiplanar external fixation, open articular bony reduction, and internal fixation, as well as non-operative treatments, such as casting, along with the inclusion of all studies regardless of publication year, further allows the evaluation of the outcome of the therapeutical approaches over time, considering that the therapeutical strategies might have changed and will be the fundament of the present review.

The present systematic review with a meta-analysis sought to summarize the available evidence on clinical outcomes and complications following conservative and surgical treatment of talar fractures.

## 2. Materials and Methods

### 2.1. Study Selection

The present systematic review was performed in accordance with the Preferred Reporting Items for Systematic Reviews and Meta-Analyses (PRISMA) statement [39]. The literature was searched from inception up to 2020. The targets of the structured search approach were prospective and retrospective studies focusing on clinical outcomes of talar fractures. We applied language restrictions to obtain studies published in English or German. Additionally, a combined medical subject heading (MeSH) and free-text term search in Medline (via OVID), including the MeSH terms “Talus/”, “Tarsal bones/”, “Ankle Joint/”, and “Foot injuries/”, was performed. Truncations were used to retrieve all forms of the search terms. A combination of search terms was performed by the Boolean operators AND and OR (Table 1).

We included all clinical studies in humans with prospective and retrospective study designs, assessing outcomes of non-operative and operative talar fractures with a follow-up of at least six months. After initially including all relevant studies, exclusion criteria were applied as follows:

1. Reviews/meta-analysis
2. Case reports
3. Fractures not involving the talar body, neck, or head
4. Non-human studies
5. Non-German or English studies
6. Follow-up under six months

### 2.2. Data Extraction and Organization

The selection of studies was performed by two independent reviewers (R.R. and J.Z.) in a two-step process. Titles and abstracts were first screened for relevance, followed by a full-text analysis. Data extraction was based on a standardized data extraction form that included all relevant information:

1. Name of the author(s), name of the journal, year of publication, study type, level of evidence
2. Fracture localization (neck, body, head)
3. Number of patients and the examined talus fractures stratified by fracture location
4. Epidemiological data (age, sex)
5. Follow-up in months (average and range)
6. Number of patients in follow-up/dropouts
7. Number of patients with open fractures
8. Number of patients treated surgically
9. Time until surgery in days
10. Postoperative readjustment of the fractures (postoperative reduction)
11. Poor or no fracture healing (malunion/non-union)
12. Type of surgical treatment
13. Frequency of early complications
14. Fracture classification
15. Number of patients with the respective degree of fracture severity, according to classification
16. Treatment result (result of the specific outcome scores)
17. Complications (AVN, osteoarthritis)
18. Frequency of joint stiffening (arthrodesis)

The results of the specific outcome scores were extracted, and the number of patients with excellent, good, moderate, and bad results was noted. Total numbers of patients with AVN and posttraumatic osteoarthritis signs were extracted regardless of severity. The number of patients with excellent and good results according to the individual utilized outcome scores were then pooled to determine the proportion of successful interventions in each cohort. The proportions of patients with AVN and posttraumatic osteoarthritis were noted to determine the proportion of complications in each cohort.

### 2.3. Assessment of the Methodological Quality of Included Studies

The methodological quality of included studies was assessed with the Coleman Methodology Score (CMS) [40]. The Coleman Methodology Score is a helpful tool for quantifying the quality of clinical studies. Ten items are used to evaluate the methodology of a study. The CMS consists of two parts (A and B) (Table 2). The subcategories in part A are assessed individually (maximum 60 points). In part B, multiple points can be awarded per subgroup (maximum 40 points). The sum of both parts results in a value between 0 (worst methodological quality) and 100 (best methodological quality). Furthermore, we determined the level of evidence for each of the included studies [41]. The following evidence levels were applied: level 1, randomized controlled study; level 2, prospective cohort study; level 3, case control study; level 4, case series; and level 5, expert opinion.

### 2.4. Statistics

We included all studies matching our predefined inclusion and exclusion criteria in the qualitative synthesis results. In the first step, the studies were stratified into subgroups depending on their fracture localization. Extracted outcome scores were used to determine the proportion of successful interventions and complications within each cohort. Hereafter, we performed a meta-analysis of each subgroup to assess and compare clinical outcomes of studies within each subgroup. A proportion meta-analysis using a random effects model considering the weights of individual studies for outcome calculations was performed with the meta package in R (R studios, version 3.6.2). Heterogeneity was assessed with *I*^2^ statistics. Values of *I*^2^ more than 25%, 50%, and 75% were defined as low, moderate, and high heterogeneity, respectively [42]. Meta-analyses and their graphic representation using forest plots were implemented using the metaprop and forest commands [43].

## 3. Results

### 3.1. Study Characteristics and Qualitative Outcome Assessment

**Figure 1:**
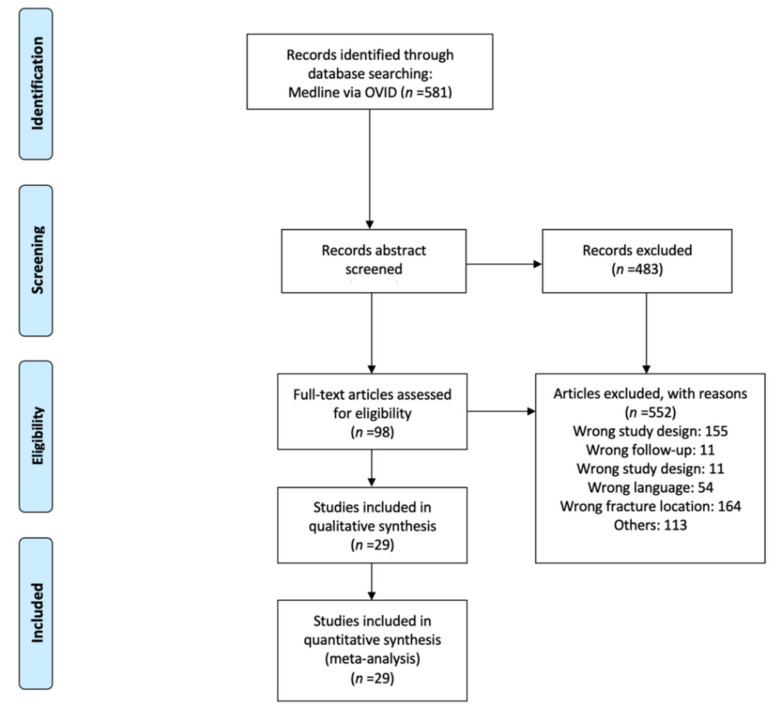
PRISMA flow diagram of the selection process.

A total of 581 studies were identified through the database search. After screening the abstracts, 98 studies remained for full-text analysis. Overall, *n* = 552 studies were excluded, mainly because the predefined study design criteria were not fulfilled (*n* = 155) or the fracture localization was not of interest (*n* = 64). Finally, 29 publications matching the eligibility criteria were included in the qualitative synthesis (Figure 1) [4,6,16,17,18,23,28,29,35,44,45,46,47,48,49,50,51,52,53,54,55,56,57,58,59,60,61,62,63]. All of these studies provided outcomes with sufficient extractable quantitative data, and could be included in the quantitative synthesis.

The 29 included studies included 1193 talar neck, body, and head fractures. On average, a study subgroup consisted of 41.1 (6–137) talar neck, body, and/or head fractures. The mean age across all study groups was 33.35 ± 5.5 years. At the time of treatment, the youngest patient was seven, and the oldest was 83 years old. The gender distribution could be determined for 27 studies, including 1050 patients (88%). In these studies, 767 (73%) patients were male and 283 (27%) were female. A mean follow-up period of 50 months could be determined from the data provided in 27 studies. The included studies were published between 1967 and 2019. Characteristics of the included studies are shown in Table 3.

Eight studies examined talar neck and corpus fractures [16,17,18,44,48,50,52,54,59], whereas eleven studies solely focused on talar neck fractures [4,6,28,29,47,51,52,55,56,61,62]. A more detailed subgroup analysis was carried out in 17/29 included studies. Consequently, fractures were classified according to their location and severity, and their respective clinical outcome was examined. Talar neck/talar body fractures and talar neck fracture subgroups were formed to investigate AVN and posttraumatic OA as complications. Another subgroup analysis was performed for patients suffering osteoarthritis with subsequent arthrodesis as a follow-up intervention.

A follow-up examination was carried out for 987 fractures (82%). In 325/987 (32%) fractures, the result was judged excellent; in 257/987 (26%) fractures, the result was good, and 216/987 (21%) of the fractures showed moderate results in the clinical follow-up examination. A total of 188/987 (19%) fractures showed unsatisfactory results in the follow-up examination. All 29 included studies provided information on complications. Complications were found in 741/1184 (62%) cases. Of these, 314 cases were OA, requiring *n* = 81 arthrodesis and *n* = 18 subtalar fusions as follow-up interventions.

A total of 994/1184 patients from 26/29 studies could be examined depending on the fracture location. From these, 803/994 (80%) had talar neck fractures, 153/994 (15%) talar body fractures, and 40/994 (5%) talar head fractures. Moreover, 151/919 (16%) open fractures were found in 20/29 included studies providing detailed information about the fractures and concomitant injuries.

In 17/29 studies, fractures were classified according to location and severity of the fracture. Hawkins 1 and Hawkins 2 fractures were seen in 219 patients, whereas Hawkins 3 and 4 were seen in 133 patients. Furthermore, *n* = 29 and *n* = 31 fractures were judged as Marti/Weber 1 + 2 and 3 + 4 grade, respectively. For Szyszkowitz classification 1 + 2 and 3 + 4, the respective numbers were *n* = 36 and *n* = 59.

### 3.2. Assessment of the Methodological Quality

All included studies were retrospective studies. The majority of studies were case series corresponding to level 4 evidence. The mean CMS A was 23.9 (range: 9–36), whereas the mean CMS B was 10.4 (range: 8–13). The corresponding total mean CMS was 34.3 (range: 19–4), indicating a generally moderate methodological quality of the included studies (Table 4).

### 3.3. Quantitative Analysis

#### 3.3.1. Success Rates

**Figure 2:**
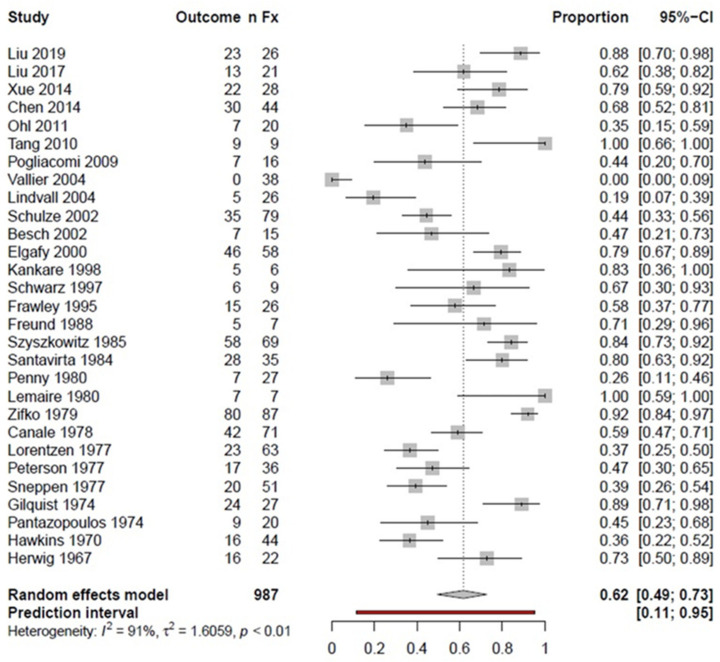
Forest plot showing the proportion meta-analysis of pooled talar fractures; *n*Fx: number of fractures; CI: confidence interval.

The success rate (i.e., good or excellent outcome) for pooled fractures regardless of localization was 62% (Figure 2). A subgroup analysis based on the fracture location resulted in a 60% success rate for TN fractures and a 50% success rate for TN/TB fractures. However, statistical heterogeneity was high, with *I*^2^ values of 91%, 85%, and 92% for pooled fractures, TN, and TN/TB, respectively.

**Figure 3:**
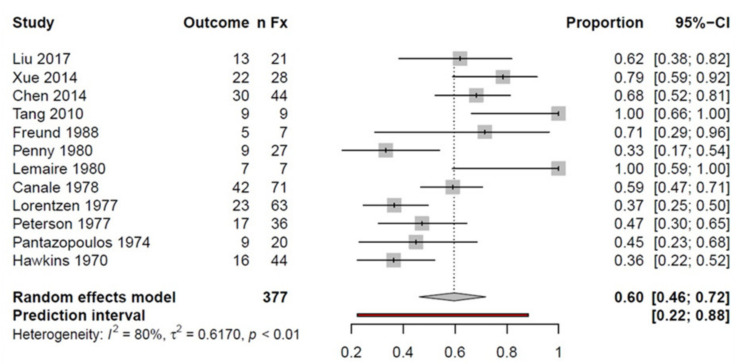
Forest plot showing the proportion meta-analysis of talar neck fractures; *n*Fx: number of fractures; CI: confidence interval.

Twelve studies were considered in the TN fracture subgroup, including a total of 377 fractures. Overall, 60% of the examined fractures had a good or excellent outcome in the follow-up examination, regardless of the fracture severity (Figure 3).

**Figure 4:**
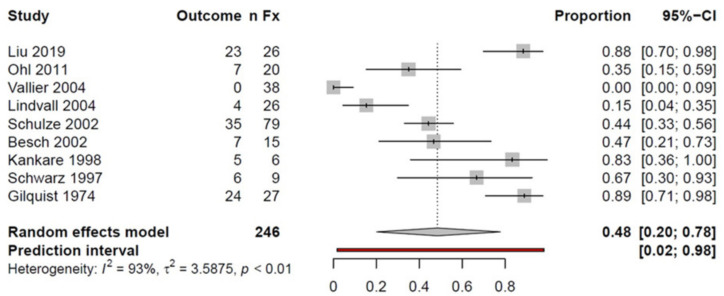
Forest plot illustrating the proportion meta-analysis of talar neck and body fractures; *n*Fx: number of fractures; CI: confidence interval.

The subgroup TN/TB fractures included *n* = 246 fractures with a 48% success rate, indicating a good or excellent outcome in nearly half of the patients (Figure 4).

A subgroup analysis was carried out according to fracture location and severity. The fractures classified according to Hawkins 1 (*n* = 69) showed a 65% success rate. The heterogeneity in the subgroup was judged low, with *I*^2^ = 27%. Hawkins 2 fractures (*n* = 150) revealed a success rate of 48%, with a moderate heterogeneity of *I*^2^ *=* 47%. Similarly, Hawkins 3 fractures (*n* = 124) comprised success rates of 40%, with a moderate heterogeneity of *I*^2^ *=* 48%. In contrast, Hawkins 4 (*n* = 9) fractures were associated with low success rates of 25%, with no observable heterogeneity (*I*^2^ *=* 0%).

In two studies [46,60], talar fractures were classified using the Szyszkowitz classification. Success rates stratified by the Szyszkowitz classification were 91% for type 1 (*n* = 11; *I*^2^ = 0%), 77% for type 2 (*n* = 25; *I*^2^ = 64), 79% for type 3 (*n* = 20; *I*^2^ = 54%), and 62% for type 4 (*n* = 39; *I*^2^ = 59%).

Two studies [16,59] including 13 fractures of the talar body demonstrated a success rate of 56% (*I*^2^ = 65%). Schulze et al. [18] classified fractures according to Marti/Weber. Success rates stratified by the Marti/Weber talus fracture classification were 53% for type 1 (*n* = 15), 57% for type 2 (*n* = 14), 44% for type 3 (*n* = 32), and 26% for type 4 (*n* = 19).

Two studies [50,54] reported outcomes of talar body fractures depending on fracture course. A success rate of 42% was found in five fractures with a coronal course and 31% in seven fractures with a sagittal course. The heterogeneity was negligible, with *I*^2^ = 17% and *I*^2^ *=* 0% for coronal and sagittal fracture courses, respectively.

#### 3.3.2. Complication Rate

**Figure 5:**
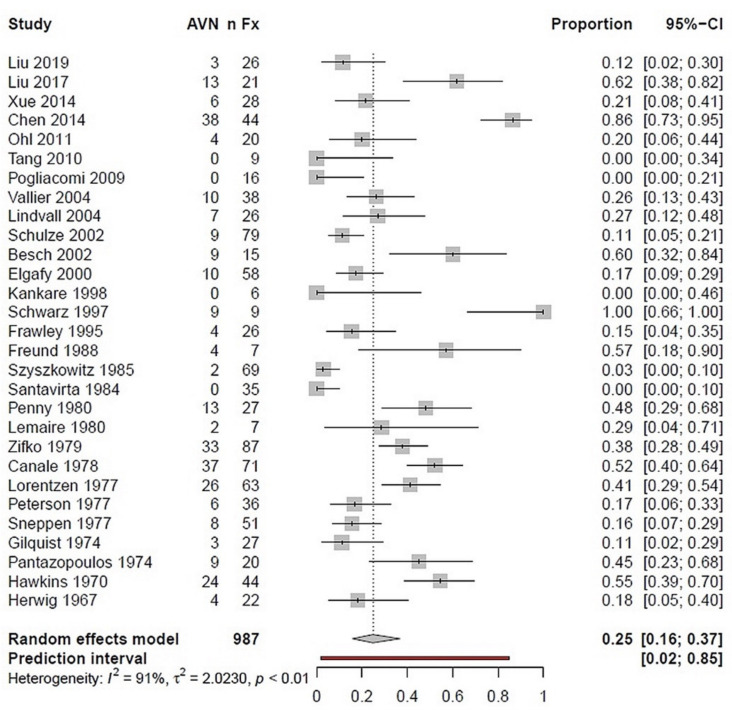
Forest plot illustrating the prevalence of posttraumatic AVN following talar fractures in a proportion meta-analysis; *n*Fx: number of fractures; AVN: avascular necrosis; CI: confidence interval.

All 29 included studies were considered for evaluation of avascular bone necrosis (AVN) as a complication. AVN had a prevalence of 25% in a total of 987 talus fractures, regardless of the severity of the fracture. Notably, heterogeneity was very high, with an *I*^2^ value of 91% (Figure 5). Another subgroup analysis on talar neck fractures (*n* = 377) revealed a 43% necrosis rate (*I*^2^ = 85%). Nine studies reported TN/TB fractures with additional information on AVN. In this subgroup, including *n* = 246 fractures, a necrosis rate of 25% was estimated (*I*^2^ = 88%).

**Figure 6:**
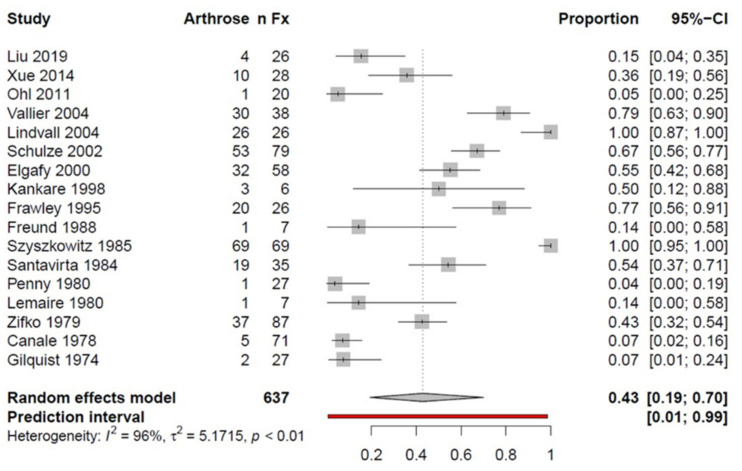
Forest plot illustrating the prevalence of posttraumatic osteoarthritis following talar fractures in a proportion meta-analysis; *n*Fx: number of fractures; AVN: avascular necrosis; CI: confidence interval.

Data from 17 studies investigated posttraumatic osteoarthritis (OA) following talus fractures. An OA rate of 43% was determined by analyzing *n* = 637 fractures. As for AVN analysis, the *I*^2^ value was 96%, and thus indicated high heterogeneity (Figure 6).

## 4. Discussion

The present systematic review and meta-analysis aimed to investigate the clinical outcomes of talar fractures, with a special focus on success as well as complications. Based on the available evidence, clinical outcomes of talar fractures were dependent on their severity, and were generally unsatisfying. We estimated a success rate of 62% for 987 fractures, regardless of the location and severity of the fracture. In 12 studies including *n* = 377 fractures of the talar neck, the success rate was 60%. Furthermore, the AVN rate was estimated to be 25% for pooled talar fractures, with higher rates (43%) for talar neck fractures than talar neck and body fractures (25%). We also found similar rates of posttraumatic osteoarthritis (43%).

### 4.1. Pooled Talar Fractures

When pooling all talar fractures (*n* = 987) in the present meta-analysis, a success rate of 62% was found in conjunction with high heterogeneity of *I*^2^ = 91%, questioning the reliability of the results. Comparable studies examining talar fractures are currently scarce. Halvorson et al. (2013) [33] reported a success rate of 56% in 429 talar neck fractures. Sneppen et al. (1977) [35] estimated a 39% success rate for 51 fractures of the talar body. Similarly, Dumont et al. (2007) [64] examined the clinical results of 41 talar fractures, and reported an average success rate of 39%.

### 4.2. Talar Neck Fractures

Twelve studies were included in the evaluation of the outcome of talar neck fractures. The success rate was 60% for the 377 fractures. Halvorson et al. examined outcomes of talar neck fractures in 2013 [33]. Results from a total of 943 fractures of the talar neck were collected from 21 studies. Similar to the present meta-analysis, not all fractures could be clinically reexamined due to loss to follow-up, and remaining fractures were reexamined using a wide variety of outcome scores. Nonetheless, the authors were able to extract the clinical results of 429/943 fractures, which were examined using the Hawkins score. A success rate (i.e., excellent and good results) of 56% was demonstrated. A total of 87 patients had an excellent result, and 152 revealed good results in the follow-up examination. The results are in accordance with the results provided in the present work.

### 4.3. Fracture Severity

Several classifications have been established to determine the severity of talar fractures. Outcomes of the 29 included studies were described with eight different score systems: 11 × Hawkins score [4], 6 × AOFAS score [65,66], 1 × Ankle-Hindfoot Scale (AHS) [66,67], 5 × Maryland Score [68], 1 × Foot Function Index (FFI) [66], 1 × Mazur Score [68], 1 × Kiel Foot Score [44], and 1 × score according to Frawley [46]. All mentioned score systems share the following characteristics: they measure the outcome after talar trauma based on the range of motion, strength, functionality, stability, and pain. The Hawkins classification, modified by Canale and Kelly, is most often used for talar neck fractures. However, other classifications (including the Szyszkowitz classification and Marti/Weber) are also commonly found in the literature. A shared feature of these gradings is that they affect the treatment and prognosis of the fractures, as both depend on the severity of the fracture. In all classifications, non-dislocated fractures are considered to result in fewer complications, whereas fractures with complete dislocation or fragmented fractures are more often associated with a complicated course, as demonstrated in the present work.

In 69 fractures classified according to Hawkins 1, a success rate of 65% was found. In contrast, the success rate for nine fractures that fell into the Hawkins 4 classification was 25%. The heterogeneity in this subgroup analysis lies in a range of 0–48%, and can therefore be classified as insignificant to moderate. The estimation results can thus be viewed as relatively consistent. However, the number of fractures in the individual subgroups was inconsistent. In the largest group (Hawkins 2), there were 150 examined fractures. In contrast, only nine fractures were included in the Hawkins 4 fracture group. A similar picture emerges when focusing on the fracture outcomes classified according to Marti/Weber and Szyskowitz. In summary, despite the low number of patients in the individual groups, there is a clear tendency towards higher success rates for simple fractures versus low success rates for complex fractures.

### 4.4. Complications

All 29 inclusion studies could be included to evaluate the avascular bone necrosis (AVN) rates following talar fractures. An AVN rate of 25% was found in a total of 987 talus fractures, regardless of the severity of the fracture. In the subgroup of fractures of the talar neck (n = 377), the result corresponds to a 43% AVN rate. Fractures of the talar neck and body were examined in nine studies. In this subgroup with *n* = 246 fractures, a necrosis rate of 25% was determined. Although there was a considerable amount of heterogeneity among these studies, the results obtained largely coincide with those provided by Dodd et al. [36]. They evaluated the data of 26 studies with a total of 980 talar neck fractures, and determined an overall AVN rate of 31%. Similarly, Metzger et al. [11] reported an AVN rate of 37% in 12 studies reporting a total of 589 talar neck fractures.

Information on posttraumatic osteoarthritis was obtained from 17 studies, including 637 fractures that were identified in 43% of all cases, regardless of fracture location and/or severity. Dodd et al. [36] examined degenerative changes in the subtalar joint of 647 talar fractures. They reported a posttraumatic osteoarthritis rate of 49%. This result is in accordance with the results determined in the present work. In contrast, Halvorson et al. [33] reported posttraumatic osteoarthritis rates of 68% in 635 talar neck fractures. The tibiotalar, subtalar, and talonavicular joints were affected. In some cases, multiple joints were involved at the same time. Notably, the subtalar joint was most often affected. Sanders et al. [67] examined 70 fractures of the talar neck in 69 patients and concluded that the initial functional results greatly predicted the follow-up complication rates. The incidence of secondary reconstructive surgery after talar neck fractures increased over time, and was most commonly performed to treat subtalar arthritis or malalignment after inadequate fracture healing. The calculated percentages of patients who needed secondary surgery at one, two, five, and 10 years were 24%, 32%, 38%, and 48%, respectively.

### 4.5. Strengths and Limitations

The present work is associated with strengths and limitations. First, the Hawkins classification [4] modified by Canale and Kelly [6] is the most common classification system for talar neck fractures [69,70]. Drummond Filho et al. showed that the inter- and intra-rater reliability of the Hawkins classification was generally satisfactory [71]. However, no reliability studies are currently available for the other classifications considered in the included studies. Another limitation when comparing the included studies was the variety of different outcome scores. We pooled excellent and good results to estimate success rates. Thus, the difference between the scoring systems could be partially counteracted. The two most frequently used outcome scores in the included studies were the Hawkins score and the AOFAS Ankle-Hindfoot score. As far as we know, none of these scoring systems have yet been evaluated with regards to their validity and/or reliability in patients with talar neck fractures. The AOFAS score is one of the most frequently used outcome measures in foot and ankle surgery, although it has not yet been adequately tested regarding validity or reliability. This remains a problem when assessing the outcome of foot and ankle trauma. Overall, comparing patient outcomes is difficult without reliable and standardized outcome measures [36]. Unfortunately, we found no studies comparing the scoring systems mentioned above. An in-depth analysis of the sources of heterogeneity using meta-regression techniques did not allow any meaningful conclusion. According to the Cochrane handbook, this would be defined as heterogeneity that cannot be explained [72]. Therefore, we performed a meta-analysis considering the heterogeneous character of the studies by applying a random effects model, following the recommendations of the Cochrane handbook. A random effects model, in contrast to the fixed effects model, accounts for statistical heterogeneity. The authors assume that the factors of the surgeon and therapy could be the most straightforward explanation, as the publications ranged from 1974–2019, and, in this time frame, the experience of the surgeons and the development of the surgical technique might have a significant influence that, however, cannot be analyzed statistically in the present study.

Furthermore, the included studies of the present work revealed a rather moderate methodological quality, with a mean CMS of 34.3 (range: 19–47). None of the included studies achieved the maximum possible number of points. Most studies reached only a few points in the categories study size, study type, and description of the postoperative rehabilitation protocol. The Coleman Methodology Score was developed by Coleman et al. to assess the methodological quality of clinical studies. Since then, the score has been used in numerous studies dealing with trauma surgery and orthopedics. In this systematic review, we applied the CMS for the first time to evaluate the methodological quality of studies focusing on talar fractures. As most included studies showed a level 4 grade of evidence, selection bias could have been a problem. However, most included studies reported that patients were consecutively included. The selection process and the handling of excluded patients are assessed in the Coleman Methodology Score in part B3. The included studies received an average of 3.5 out of a maximum of 15 possible points. Therefore, selection bias should be considered when interpreting the present results. Comparable systematic reviews on this topic were published between 2013 and 2017, but mainly focused on talar neck fractures [33,36,73]. The majority of the currently available reviews call for an analysis of predictive factor and subgroup evaluations to examine the reason for the high complication rates. The advantage of our initial study protocol compared to the available reviews was the focus on talar fractures generally, and not only talar neck fractures. On the one hand, this broadens understanding and allows comparability of complication rates in different talar regions within one systematic review protocol. On the other hand, we have applied a random effects model while analyzing the studies, which better accounts for the heterogeneous character of the studies compared to the current meta-analysis conducted on talar neck fractures previously [36]. A constant evaluation of the literature on this topic in the form of a systematic approach is warranted to find new and promising developments and therapeutic advances. Our literature search yielded one recent study performed by Lui et al., which generally showed lower AVN and osteoarthritis rates than those obtained by the pooled prediction in the random effects model [53]. After anatomical reduction, they used two screws and fixed the lateral malleolus with distending wires. This recent reporting indicated that internal fixation of talar fractures with lateral malleolar osteotomy might be associated with satisfying clinical outcomes. Most of the evidence included in the aforementioned systematic reviews was published in the last century, and might not be comparable to the currently performed techniques. Historically, therapy of talar fractures was generally performed more conservatively compared to the current strategies. Thus, prospective studies and literature searches should be performed in regular time intervals so as not to miss any progress on this topic that might be of relevance for the therapy of future affected patients.

### 4.6. Recommendations

Overall, the current evidence on clinical outcomes following talar fractures must be considered insufficient. To the best of our knowledge, there are no randomized controlled trials on this topic, limited to the current evidence grade. Prospective randomized and nonrandomized studies will be of help to gain more reliable evaluations of outcomes in future. A standardization of talar fracture classifications and scoring systems would improve the comparability of future studies. Large sample-sized prospective studies are warranted to detect further predictive factors influencing the currently unsatisfactory clinical outcome of patients undergoing talar fracture treatment. Specifically, the confounding factors of the surgeon and exact treatment strategy should be considered in future studies. Studies involving multiple surgeons, secondary treatments, and/or multiple treatments bias the result evaluations, and might be the reason for the current heterogeneity in the literature. Considering the remaining high rate of complications despite the development of diagnostics and surgical therapies, delayed surgery and remaining fracture displacements might be the main risk factors for complications. However, current reports do not call for immediate emergent surgical management using open reduction and internal fixation in contrast to historical recommendations [74]. Nevertheless, nonsurgical treatment should only be reserved for nondisplaced fractures. Nearly 95% of the talar neck fractures since 2000 were treated surgically [36]. Notably, the quality of reduction seems to be more important than the timing of reduction [75]. A dual incision technique utilizing anteromedial and anterolateral approaches for talar neck fractures under good visualization and fluoroscopy during reduction helps to prevent rotational and angular malreductions [76]. For treatment of talar neck fractures, there is no clear clinical evidence for superiority between screw fixation alone and screw/plate fixation [76]. However, screw fixation may be advantageous for maintaining the talar neck’s crucial blood supply. For talar body fracture treatment, the biplanar chevron technique showed high malunion rates of up to 30% when fixed with two lag screws compared to buttress plates, whereas, for talar head fractures, a dual incision technique with medial-to-lateral screws recessed into the subchondral bone was recently introduced for fixation [77]. The following key points should be considered by orthopedic surgeons and researchers, which might help to decrease the complication rates in the future: (1) standardized treatment algorithms based on high-evidence studies; (2) standardized outcome evaluations of the applied therapies; (3) timely soft tissue coverage and management for open fractures; and (4) the quality of the surgical technique seems to be more important than the time of reduction.

## 5. Conclusions

The present meta-analysis on clinical outcomes of patients undergoing conservative or surgical treatment for talar fractures reveals a lack of reliable prospective evidence. Talar fractures are associated with relatively poor postoperative outcomes, high rates of AVN, and posttraumatic osteoarthritis. Poor outcomes revealed a positive association with fracture severity. Prospective studies investigating predictors for treatment success and/or failure are urgently needed to improve the overall quality of life and function of patients undergoing surgical treatment due to talar fractures.

## Figures and Tables

**Table 1:** Search strategy.

Search Step	Search Terms and Operators
1	Talus/
2	Tarsal bones/
3	exp Tarsal joints/
4	Ankle Joint/
5	Foot injuries/
6	1 or 2 or 3 or 4 or 5
7	Fractures, Bone/
8	6 and 7
9	talus.tw./
10	8 and 9
11	fracture * tw./
12	10 and 11
13	exp animals/not humans/
14	12 not 13

* Word truncation.

**Table 2:** Coleman Methodology Score.

Step	Criterion	Subcriterion	Score
Coleman Methodology Score Part A (Max. 60 points)			
1.	Study size	>60	10
		41–60	7
		20–40	4
		<20 or no information	0
2.	Mean follow-up (in months)	>24	5
		12–24	2
		<12, no information or unclear	0
3.	Number of different surgeries performed for each outcome included	Only one procedure performed	10
		>1 procedure performed, but used in at least 90% of patients	7
		No information, unclear, or use of only one procedure in less than 90% of the patients included	0
4.	Type of study	Randomized controlled	15
		Prospective cohort	10
		Retrospective cohort	0
5.	Diagnostic certainty (use of sonography, MRI, or histopathology to confirm the diagnosis)	100% of patients	5
		>80% of patients	3
		<80% of patients	0
6.	Description of the surgical therapy procedure	Adequate (surgical technique mentioned and described in detail)	5
		Moderate (surgical technique mentioned, but no detailed description)	3
		Inadequate (surgical technique not mentioned) or unclear	0
7.	Description of postoperative rehabilitation	Well-described and > 80% of patients compliant to therapy	10
		Well-described and 60–80% of patients compliant to therapy	5
		No protocol described or < 60% of patients compliant to therapy	0
**Coleman Methodology Score Part B (Max. 40 points)**			
1.	Outcome criteria (0 points are awarded in this section if the outcome criteria are not defined precisely)	Clearly defined measures for outcome	2
		Follow-up examination and evaluation of the outcome clearly defined	2
		Use of outcome criteria with good reliability	3
		Use of outcome criteria with good sensitivity	3
2.	Procedure for outcome-assessment	Patients called in for a follow-up examination	5
		Examiner independent of the surgeon	4
		Assessment is documented in writing	3
		Completion of the assessment with minimal involvement of the examiner	3
3.	Description of patient selection	Selection criteria specified and unbiased	5
		Recruitment rate > 80%	5
		Recruitment rate < 80%	3
		Exclusion criteria clearly described or 100% recruitment rate	5

**Table 3:** Study characteristics.

Author and Year	Study Design	Localization	NoFx	NoSFx	Conservative or Closed Fixation	ORIF	External Restraint	Other Primary Intervention
Liu 2019	Retrospective	TN, TB	26	26				23
Liu 2017	Retrospective	TN	22	21		21		
Xue 2014	Retrospective	TN	31	22	9	22		
Chen 2014	Retrospective	TN	44	38		38		
Ohl 2011	Retrospective	TN, TB	20	20		20		
Tang 2010	Retrospective	TN	9	9				9
Pogliacomi 2009	Retrospective	TN, TB, TH	16	13				NR
Vallier 2004	Retrospective	TN, TB	56	56		37		19
Lindvall 2004	Retrospective	TN, TB	26	26	NR	7		
Schulze 2002	Retrospective	TN, TB	80	80		67	1	12
Besch 2002	Retrospective	TN, TB	23	23		22	5	4
Elgafy 2000	Retrospective	TN, TB	60	48				
Kankare 1998	Retrospective	TN, TB	6	6		8		
Schwarz 1997	Retrospective	TN, TB	19	9		5		
Frawley 1995	Retrospective	TN, TB, TH	28	20		20		
Freund 1988	Retrospective	TN	7	7	2	3		2
Szyszkowitz 1985	Retrospective	TN, TB	85	85	23	54		8
Santavirta 1984	Retrospective	TN, TB, TH	35	11		7		4
Penny 1980	Retrospective	TN	40	30		NR		9
Lemaire 1980	Retrospective	TH	7	7		7		
Zifko 1979	Retrospective	TN, TB, TH	137	24	NR	NR		NR
Canale 1978	Retrospective	TN	71	57	NR	25		11
Lorentzen 1977 E	Retrospective	TN	123	20	33	14		2
Peterson 1977	Retrospective	TN	46	29	14	7		8
Sneppen 1977	Retrospective	TN	51	9		9		
Gilquist 1974	Retrospective	TN, TB	28	13	9	13		
Pantazopoulos 1974	Retrospective	TN	20	11	5	10		1
Hawkins 1970	Retrospective	TN	55	34	24	22		
Herwig 1967	Retrospective	TN, TB, TH	22	13		9		4

NoFx: number of fractures; NoSFx: number of surgically treated fractures; ORIF: open fixation or open reduction with internal fixation; TN: talar neck; TB: talar body; TH: talar head; NR: not reported.

**Table 4:** Assessment of the methodological quality of included studies.

Author	Study Year	Study Design	Level of Evidence	CMS A	CMS B	CMS Total
Liu	2019	Retrospective	4	21	12	33
Liu	2017	Retrospective	4	29	12	41
Xue	2014	Retrospective	4	34	13	47
Chen	2014	Retrospective	3	24	12	36
Ohl	2011	Retrospective	4	36	11	47
Tang	2010	Retrospective	4	10	9	19
Pogliacomi	2009	Retrospective	4	17	10	27
Vallier	2004	Retrospective	4	30	8	38
Lindvall	2004	Retrospective	4	24	12	36
Schulze	2002	Retrospective	4	30	12	42
Besch	2002	Retrospective	3	24	10	34
Elgafy	2000	Retrospective	4	25	10	35
Kankare	1998	Retrospective	4	28	10	38
Schwarz	1997	Retrospective	4	26	10	36
Frawley	1995	Retrospective	4	24	10	34
Freund	1988	Retrospective	4	20	10	30
Szyszkowitz	1985	Retrospective	4	27	10	37
Santavirta	1984	Retrospective	4	9	10	19
Penny	1980	Retrospective	4	14	10	24
Lemaire	1980	Retrospective	4	22	10	32
Zifko	1979	Retrospective	4	25	10	35
Canale	1978	Retrospective	4	25	10	35
Lorentzen	1977	Retrospective	4	22	10	32
Peterson	1977	Retrospective	4	27	10	37
Sneppen	1977	Retrospective	4	22	10	32
Gilquist	1974	Retrospective	4	29	10	39
Pantazopoulos	1974	Retrospective	4	24	10	34
Hawkins	1970	Retrospective	4	22	10	32
Herwig	1967	Retrospective	4	24	10	34

## Data Availability

The data that support the findings of this study are available from the corresponding author, upon reasonable request.
